# The impact of the Dementia Care in Hospitals Program on hospital acquired complications – a non-randomised stepped wedge hybrid effectiveness-implementation study

**DOI:** 10.1186/s12877-024-05548-3

**Published:** 2024-12-02

**Authors:** Mark W. Yates, Kasia Bail, Sean MacDermott, David Skvarc, Meredith Theobald, Michelle Morvell, Jessica C. Jebramek, Ian Tebbut, Brian Draper, Henry Brodaty

**Affiliations:** 1 Grampians Health, Ballarat Health Services, School of Medicine, Faculty of Health, Deakin University, Ballarat, VIC Australia; 2 Ageing Research Group, Faculty of Health, Synergy Nursing and Midwifery Research Centre ACT Health, University of Canberra, Canberra, Australia; 3 John Richards Centre for Rural Ageing Research, La Trobe Rural Health School, Wodonga, VIC Australia; 4 School of Psychology, Faculty of Health, Deakin University, Melbourne, Vic Australia; 5 Grampians Health, Ballarat Health Services, Ballarat, Australia; 6 Health Services Data Analyst, RooCube Pty Ltd, Melbourne, Australia; 7 Professor (Conjoint), Discipline of Psychiatry and Mental Health, UNSW Medicine and Health, University of NSW, Sydney, Australia; 8 Centre for Healthy Brain Ageing (CHeBA), Discipline of Psychiatry and Mental Health, University of New South Wales, Sydney, NSW Australia

**Keywords:** Cognitive impairment, Delirium, Adverse events, Hospitals, Trial, Effectiveness, Multi-modal interventions

## Abstract

**Background:**

Hospitalized older patients with cognitive impairment (CI) experience poor outcomes and high rates of hospital acquired complications (HACs). This study investigated the effectiveness of a multimodal hospital CI identification and education program.

**Method:**

A prospective stepped-wedge, cross-sectional, continuous-recruitment, hybrid effectiveness-Implementation study was conducted in acute hospitals in four Australian states/territories. The intervention, the Dementia Care in Hospitals Program (DHCP) provided: clinical/ non-clinical hospital staff CI awareness support and education; CI screening for older patients and a bedside alert—the Cognitive Impairment Identifier (CII). The primary outcome was change in the rate of the combined risk of four HACs (urinary tract infection, pneumonia, new onset delirium, pressure injury).

**Results:**

Participants were patients aged 65 years and over admitted for 24 h or more over a 12-month period between 2015–2017 (*n* = 16,789). Of the 11,309 (67.4%) screened, 4,277 (37.8%) had CI. HACs occurred in 27.4% of all screened patients and were three times more likely in patients with CI after controlling for age and sex (RR = 3.03; 95%CI:2.74–3.27). There was no significant change in HAC rate for patients with CI (RR = 1.084; 95%CI: 0.93; 1.26). In the intervention period the raw HAC rate for all screened patients was 27.0%, which when adjusted for age and sex suggested a small reduction overall. However, when adjusted for hospital site, this reduction in HAC risk not statistically significant (RR = 0.968; 95%CI:0.865–1.083). There was considerable interhospital variation in intervention implementation and outcomes which explains the final non-significant effect.

**Conclusion:**

For patient with CI the implementation of the DCHP did not result in a reduction in HAC rates. Education for hospital staff regarding cognitive impairment screening, care support, carer engagement and bedside alerts, using the DCHP, can be feasibly implemented in acute hospitals. Reducing high frequency HACs in older hospital patients with CI, warrants further research.

Trial Registration. The trial was registered retrospectively with the Australian New Zealand Clinical Trials Registry (ANZCTR) ACTRN12615000905561 on 01/09/2015 with 92 patients (0.8% of total sample) recruited in the baseline and none in the intervention before registration submission.

**Supplementary Information:**

The online version contains supplementary material available at 10.1186/s12877-024-05548-3.

## Key messages

- The increased risk of hospital acquired complications (HAC) in patients with cognitive impairment (CI) has been attributed to poor identification resulting in insufficient patient and carer support. There is a paucity of effectiveness studies investigating the impact of interventions to reduce the high HAC rate for patients with CI. We report the largest intervention study, of which we are aware, conducted in a real-world context.
- The Dementia Care in Hospitals Program, a multimodal intervention, consisting of hospital screening for CI, education of clinical and non-clinical staff, carer engagement and an over-bedside alert is feasible and acceptable in acute care hospitals.
- Hospitals should have screening programs to alert staff to CI and its associated higher rates of HACs, and staff education and training programs to help reduce HACs levels in older patients.

## Strengths and limitations

- This is first real-world study to investigate if a multi-modal intervention using screening, education, and a bedside alert, the CII, can reduce HAC rates in patients with CI in acute hospitals.
- The intervention implementation strategies are well described and had been tested in multiple acute hospitals supporting readiness for evaluation of patient outcome such as HAC rates.
- A large sample size (*N* = 11,390) in four major teaching hospitals in four Australian jurisdictions.
- Non-randomised study design
- The complexity of the intervention and the funding body requirements resulted in variability in implementation and outcomes across sites.
- Determination of HACs relied on staff documentation.

## Introduction

Patients with dementia who are admitted to Australian hospitals experience poor overall health outcomes [1, 2]. Compared to aged matched patients without dementia, they have more adverse events, a longer length of stay, are at greater risk of developing delirium and have higher mortality [3]. Adverse outcomes lead to higher costs of care [4, 5] and underscore the need to develop more efficient and effective models of care for hospitalised patients with dementia.

A key factor underpinning poor outcomes is the under-recognition of dementia in patients admitted to hospital. Dementia is documented in fewer than half affected patients [3, 6] and delirium is similarly under-reported [7]. A salient and quantifiable feature of delirium and dementia is the presence of cognitive impairment (CI). A lack of recognition of cognitive impairment associated with these conditions can lead to a lack of appropriate support to patients and their families [8].

Though CI is associated with a range of other unrelated conditions, recognition or detection of CI can broadly serve as an indicator of a patient’s risk of adverse outcomes [6]. Clinicians using standardised cognitive testing tools can identify CI without the need for diagnostic labels such as dementia or delirium, thus broadening the likelihood of risk identification and actions for harm minimisation. Identification of all patients with CI, using pre-emptive screening tools, could alert clinical/non-clinical staff of the need to implement strategies to mitigate potential harm.

To test this hypothesis, we used the Dementia Care in Hospitals Program (DCHP), a clinical/non-clinical staff intervention that centres on early cognitive screening, providing an opportunity for improved communication and engagement with patients and their families. The DCHP uses a bedside alert that was developed with people with dementia [9], the Cognitive Impairment Identifier (CII), supplemented by a staff training program informed by carers and people with dementia [10].The core aim of the DCHP is to reduce HACs (2) by; using routine cognitive screening, identification of those patients with CI with the bedside CII which facilitates the involvement of clinical and nonclinical staff in care, teaching staff communication strategies and requesting carer involvement.

The DCHP is an acute hospital program developed by Ballarat Health Services (BHS) in 2004 in partnership with people with dementia and their families and rolled out in 27 hospitals in the State of Victoria over a 9-year period. The results of these implementations have been incompletely published in non-peer reviewed reports and presented in conference abstracts. Previous implementations of the DCHP demonstrated improvements in staff satisfaction, carer satisfaction and better staff awareness of CI. That the DCHP could be implemented was also demonstrated by achieving target CI screening rates and the consistent use of the bedside CII. A set of pre-implementation requirements which include strategies that support implementation [11] and a staff education program [12] has been developed. Program sustainability and patient outcomes were not measured. In 2015 the Australian Government funded a national rollout of the DCHP providing an opportunity for the DCHP to be implemented in similar hospital contexts outside Victoria and measure the impact on the patient outcome of HAC rates.

Of the 16 HACs described in Australia, 12 are considered nurse modifiable or sensitive [13] indicating that modification of clinical practice, education or staffing ratios can reduce the HAC event rate [14, 15]. In patients with dementia compared to no dementia four of those 12 HACs ( Urinary Tract Infection (UTI),pneumonia, pressure area and new onset delirium) have been reported to have significantly higher relative risk in both surgical and medical patients [13]. For example, it has been found that 14.7% of surgical patients with dementia experienced UTI compared to 5.6% of patients without dementia and in medical patients delirium occurred in 4.0% compared to 1.5%. These relative risks have been identified as key indicators for older people specifically, providing a quality measure for hospital’s ‘failing to maintain’ older people, similar to the quality indicator ‘failure to rescue’ [16]. Improving staff identification of CI and increasing awareness of support strategies may reduce HACs in patients with dementia, improving outcomes and reducing length of stay. Previous interventions aimed at improving care and outcomes in patients with CI have included intensive staff education and staff awareness training [17], an approach that is resource intensive and limited by cost. Other interventions have been restricted to specific populations [18] or specific wards such as aged care units [19] and not easily transferrable to the broader hospital setting [16] where rates of delirium are as high as 64% of admissions in general medicine and 68% in surgical wards [20].

This paper reports on the impact of the DCHP on the HAC rate by measuring the change in one or more of four HACs: UTI, pressure injury, pneumonia and new delirium after the adoption of the DCHP. The primary hypothesis was that the DCHP would lead to a reduced rate of HACs in patients with cognitive impairment, resulting in improved hospitalisation outcomes.

## Methods

The study protocol and methods, have been published [10] and are summarized below.

### Study design

The intervention was conducted between June 2015 to February 2018 and is a prospective stepped-wedge, hybrid effectiveness-implementation study. This study type was chosen as a good fit for the evaluation of health service reform implementation [21]. The hybrid type best fits a Type 1 design [22]. The study focuses on implementation as measured by screening, education and CII usage rates and effectiveness as measured by the impact on HAC rates. There was limited need for adaptation of the DCHP in the 4 sites as the intervention was implemented in acute hospitals so key implementation determinants were known. The DCHP was thought to be “ready” [22] for implementation and this was optimized by consumer engagement in the DCHP design and engaging the participating hospitals in some aspects of the implementation such as the screening tool used and wards involved. The timetable (Table 1) was modified because of delays in implementation. In all sites the four data collection intervals were reduced from 12 in control to 10 weeks in intervention.

### Recruitment of sites

Four sites were recruited by Expression of Interest from Australian tertiary metropolitan and regional hospitals. The 20 medical, surgical and acute geriatric participating wards were selected by the sites according to willingness to be involved [10].

### Recruitment of population and sample size

The study population are patients aged 65 years or older or aged 50 years or older for those of Aboriginal or Torres Strait Islander status admitted to participating wards. They will be henceforth referred to as older patients and represented a subset of all patients from this cohort admitted to the participating hospitals during the study. Patients remained in the study for the duration of their acute hospital stay even if they moved to non-participating wards during the admission.

### Patient and public involvement

People with dementia and their families were involved in the development of the DCHP in 2004. There was no direct patient involvement for the data provided in this paper. The intervention to be implemented involved training for hospital staff who provided feedback about their training sessions. The wider impact evaluation investigated patient and carer reported quality of life (not published).

### Intervention

**Fig.1:**
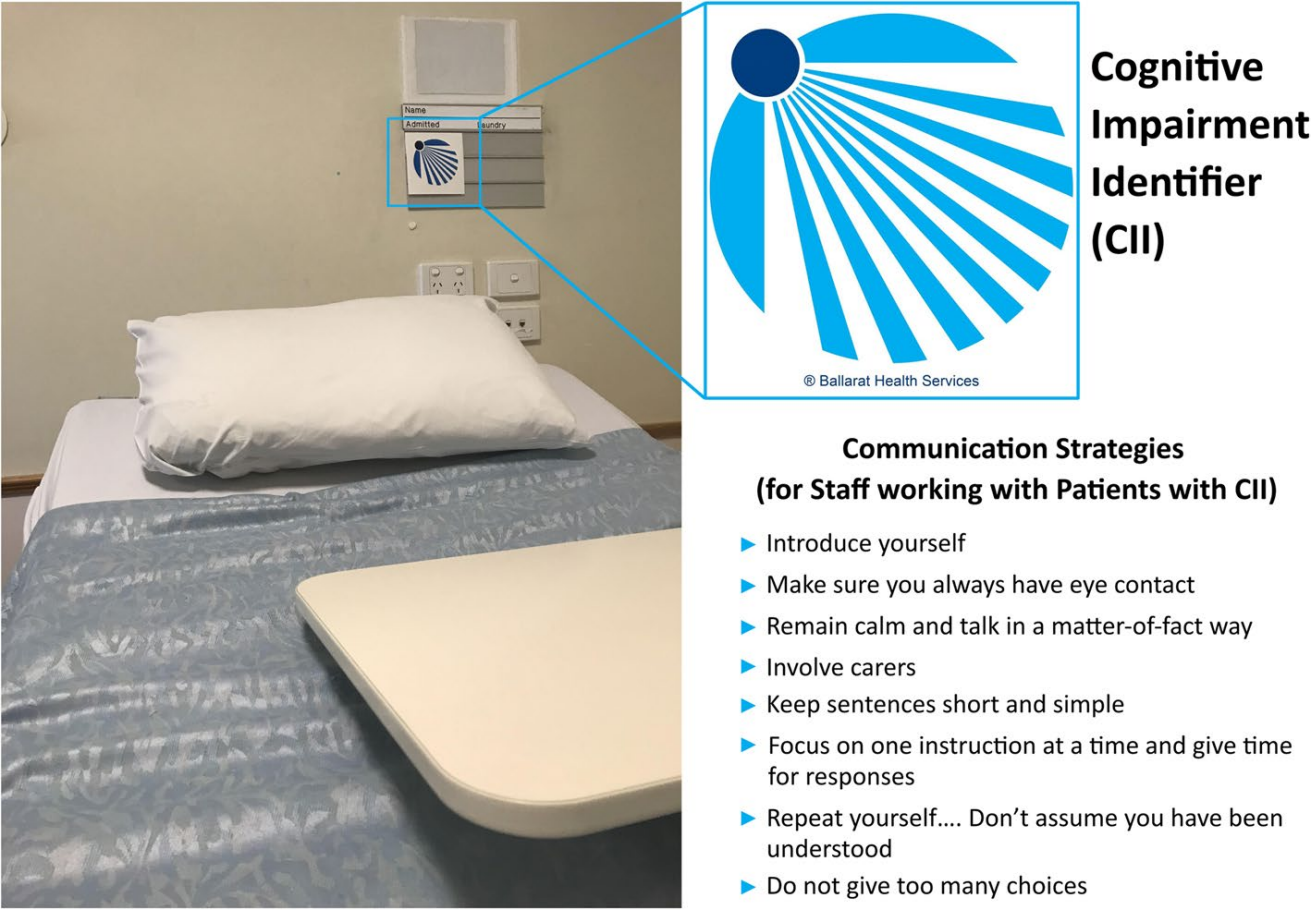
The CII and communication strategies

The DCHP intervention consists of four elements:

1. Screening the defined study population for CI, using a validated cognitive screening tool, within 24 h of admission to the ward
2. Completion of the DCHP education package by all clinical and non-clinical staff aimed at enhancing communication strategies with patients living with dementia
3. Carer and/or family engagement
4. With patient assent, placement of the CII over the bedside to alert clinical and non-clinical staff to the patients need for additional support because of CI. (Fig. 1)

In addition to these four key elements the implementation required the development of hospital specific clinical pathways (protocols, policies), executive management support, and ward based ‘champions’ for the identification and management of CI [10].

Each of the four study sites undertook the intervention in a stepped sequence of three phases with the introduction of the DCHP at each site at different time points. Phase One was normal practice and included the collection of retrospective HAC data in older patients who had cognitive impairment recorded in the hospital data report sets. This formed the basis for the power analysis. Baseline/Training (12 weeks) was used as the control period and this was followed by the intervention period of four 10 week data collection blocks. (Table 1)The control period (12-weeks) included the introduction of the screening for CI and the provision of training to a minimum of 40% of staff [10]. The start of the control period was staggered at the 4 sites over one year. In the intervention period it was expected that there would be 100% screening for CI, 80% of all staff trained and 80% use of the CII over bedsides in those who screened positive for CI was expected. The Stepped-Wedge study schedule and time periods are described in detail in the published protocol [10]. The protocol had planned for 12-week control and 48 weeks intervention. Training at sites 1 and 2 took longer than planned and the intervention period was reduced to 40 weeks to meet funding requirements.

**Table 1:**
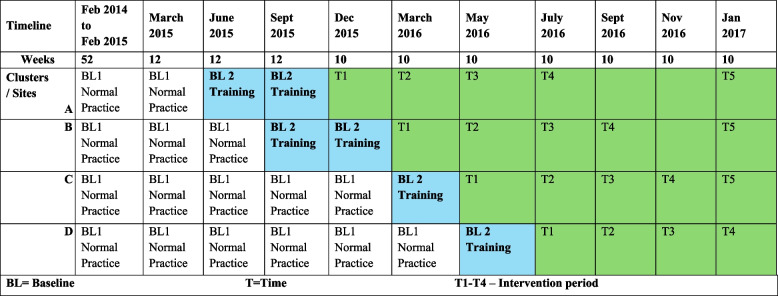
Step-wedge Timetable

*BL* Baseline, *T* Time, *T1-T4* Intervention period

Shaded cells: *BL 1* Retrospective data collection period, *BL2* the Control Period, *T1-4* the Intervention Period

### Data collection and outcomes

Data collection was undertaken during control and intervention periods. Demographic data collected across these two periods included patient specific data comprising age, sex, primary and additional Diagnostic Related Groups (DRGs), and surgical status. Episodic descriptors were length of stay and discharge ward. Cognition was screened by tests that were standard for each hospital (Supplementary Table 1).

Outcomes used to measure the fidelity of DCHP implementation were the percentage of all clinical and non-clinical staff completing the DCHP training, the percentage of cognitive screening of the study population using a validated screening tool, and the proportion of patients who screened positive for CI and had the CII placed over the bedside. Data on these outcomes were collected by audit by project managers onsite once in the control period and at four 10-week intervals (T1, T2, T3, T4) in the intervention period.

The primary outcome was the change in HAC rate in patients with CI before and after the introduction of the DCHP. It was hypothesised that an intervention that increases awareness of CI and targets support could improve the likelihood the staff would introduce known strategies prevent the target HACs. A HAC positive was defined as experiencing one or more of four selected hospital-acquired complications as coded in hospital administrative data sets based on the medical record. The four selected complications are known to be nurse modifiable. A single collective outcome was chosen to improve the power of the study. All HACs were captured only if documented as a change in condition after admission. Data on HAC rates for the study population were drawn from the Health Round Table database [23] to which all four sites contribute their coded HAC data. It was accepted that the accuracy of coding would be consistent over time.

Data for cost effectiveness, staff satisfaction/confidence caring for patient with dementia, antipsychotic usage and combined HAC rate in the total CI positive population were collected as secondary outcomes as per Table 3 in the protocol [10].

### Power calculation and analysis

The target sample size of 3750 patients was determined based on 750 positive screens for cognitive impairment per period, with calculations done at a power of 0.8 and a type I error rate of 0.05, considering clustering effects. This sample size allowed the detection of a 22.0% to 25.5% reduction in risk ratio, equivalent to a 5.0% to 5.5% reduction in absolute risk, assuming 80% power and alpha = 0.05.

### Data analysis

**Fig. 2:**
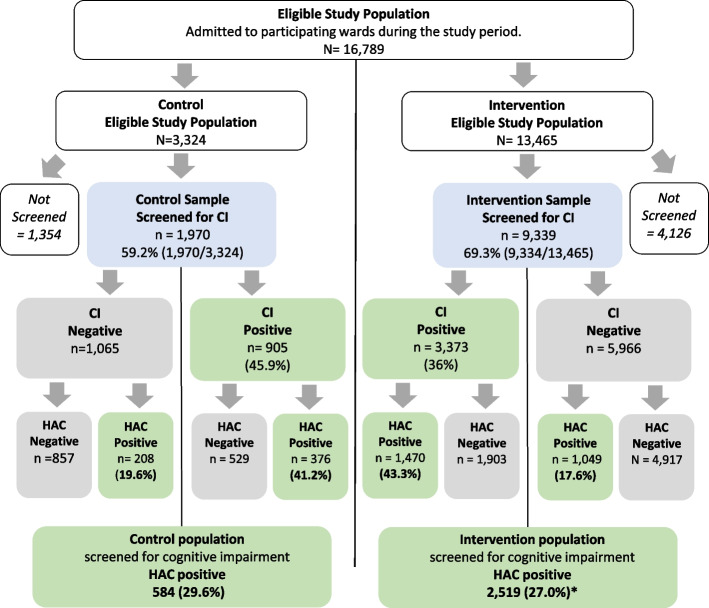
Study Population and Disposition. CI – Cognitive Impairment. HAC Positive – Experience one or more of the four target Hospital Acquired Complications. HAC Negative – Experiences none of the four target Hospital Acquired Complications * = *p* < 0.05

Rates of staff completion of the DCHP training, the screening of the study population for CI using a validated screening tool and the placement of the CII over the bedside were analyzed (Fig. 2). For the primary outcome of HAC risk, we used a log-link generalised mixed model for binary outcomes, with time as a fixed factor and hospitals as a random effect and included patient age and sex as covariates in an incomplete stepped-wedge design. Absolute outcomes were determined using a binary count rate of HACs compared across time and between hospitals. To understand intrahospital variation, the changes in HAC relative risk (RR) was compared to one of the four participating hospitals chosen as the index case (site D). Additional details are provided in our protocol [24]. For secondary analyses of within-hospital HAC rate between patients with and without CI we tested for significant differences using a two-tailed Z-test for two-samples. There was no adjustment for multiple comparisons nor age or sex for these analyses. This more straightforward approach differs from the analysis in the protocol paper [10].

## Results

### Participants

Of 47,816 older patients admitted for 24 h or longer to all four hospitals during the control and intervention periods, 16,789 (35.1%) were admitted to participating wards and comprised the **eligible study population**. Pre-study estimates for participant numbers and complication rates were met. People of Aboriginal and Torres Strait Islander status represented 2.3% of all admissions (*n* = 385) and 1.5% (*n* = 167) of the eligible population. No single hospital site contributed disproportionally to the pooled data (Supplementary Table 2); no eligible patient screened was excluded. Patients were generally balanced across sites for age, gender, and proportion of surgical patients. Differences between sites were not meaningful. (Supplementary Table 2).

The study sample was the 11,309 (67.2%) patients from the eligible study population who were screened. This comprised 1970 (59.2%) of the control and 9339 (69.3%) of intervention eligible populations respectively. The proportion of eligible patients who were screened in the control period (59.2%) was significantly less than in the intervention period (69.3%) (Fig. 2).

The study sample and the eligible population were similar in age and sex (mean age 77.5 years, SD 8.7, males 50.5%). The average age of participants across the four hospitals ranged from 78.5 to 80.5 years, SD 8.02–8.88. There was no significant age difference between sites (Supplementary Table S2).

### DCHP measures of implementation

Implementation was measured with staff training, screening rate and CII usage**.**

By study completion, of the 2,587 staff recorded as working on the wards across the four sites, 1,748 (67.6%) had received DCHP training. Nursing staff accounted for the highest proportion of staff trained (72.6%), followed by non-clinical, medical, and allied health respectively (Supplementary Table 3). The range of nursing and medical staff training in different wards varied from 50–100%.

The screening rate increased by 10.1% in the intervention period (59.2% control, 69.3% intervention), the proportion who screened positive for CI declined (45.9% control, 36.1% intervention) (Fig. 2). In both periods, those who screened positive for CI were 4.8 years older than those who screened negative.

The pooled rate of cognitive screening peaked at 72.9% during the intervention period T3 (Supplementary Table 4). The screening rates varied by hospital site, ward and time period; three sites achieved 80% or more screening in more than one time period.

The CII was displayed over the bedside for 69.8% (1907/2732) of patients with CI (Supplementary Table 5). Of the 825 patients where the CII was not used, 239 (28.9%) was associated with short stays or weekend admissions. Display of the CII was declined by 2 (1%) patients.

The proportion of patients involved in the study as a percentage of all older patients in the four hospitals was 35.1% (16,789/47816) (Supplementary Table 6).

### Change in HAC rate

Of the “all patients screened” population (11,309) 27.4% (3103) were HAC positive. Of the 4278 patients who screened positive for CI 43.1% (1846) were HAC positive. (Fig. 2).

There was no significant difference in the HAC rate after the intervention after controlling for age, sex, and CI (RR = 1.07; 95%CI: 0.922 – 1.24) (Table 2). Age was a significant predictor of HAC positivity: for every year of age the risk of HAC positivity increased by 4%, controlling for CI (RR = 1.037; 95%CI: 1.03; 1.04). Older patients who screened positive for CI were three times more likely to be HAC positive compared to those who did not have CI after controlling for age and sex (RR = 3.03; 95%CI: 2.74–3.27). In the control period, 29.6% of all older patients screened were HAC positive, however, when adjusted for hospital site, the risk of HAC was lower but not statistically significant (RR = 0.968; 95%CI: 0.865; 1.083).

**Table 2:** Percentage HAC positive patients who screened positive for CI

		Baseline		Intervention T1-T4		Total		Adjusted RR *
Hospital	Screening Result	HAC	% HAC	HAC	% HAC	HAC	% HAC	
Site A	CI	60	48.0%	246	34.6%	306	36.6%	0.57 (0.38—0.88)
Site B	CI	172	36.3%	437	43.2%	609	41.0%	1.32 (1.05—1.65)
Site C	CI	59	43.4%	336	55.8%	395	53.5%	1.66 (1.11 – 2.49)
Site D	CI	85	50.0%	451	43.0%	536	44.0%	0.79 (0.56—1.1)
Pooled	CI	376	41.2%	1470	43.2%	1846	43.1%	1.07 (0.92—1.24)

Note:* RRs are adjusted HAC rates for the patient with CI in each hospital after the intervention compared to baseline, adjusted for age and sex

In the control period, 41.5% of patient with CI experienced HACs compared to 43.2% during the intervention period (RR = 1.084; 95%CI: 0.93; 1.26). This finding was not altered after adjustment for hospital site, age, sex, or admission type. The change in HACs in the patients with CI after intervention varied across sites from –13.4% to + 12.4%. (Supplementary Table 7).

Overall, there was no detectable difference in the effect of the DCHP on the HAC positivity rate in those who screened positive for CI so the primary hypothesis was not supported. There were no important harms.

Of the secondary outcomes an improvement in staff satisfaction has been published [25]. Other secondary outcomes (Supplementary Table 8) have either are yet to be reported in peer reviewed papers but can be found in the DCHP National roll-out report [26] on the Grampians Health website [27]. Median cost per episode was lower in the intervention period for those who screened positive for CI, there was no reduction in quality of life and carer satisfaction increased.

## Discussion

This is the first study, to investigate whether HACs can be reduced by a multimodal intervention aimed at improving awareness of and communication with patients with CI. There have been other multimodal hospital based interventional studies reported but they have not measured multiple HACs as an outcome, did not use a bedside alert as part of their strategy and are often focused heavily on staff education [28–31]. This study of over 11,000 patients reports a 37.8% prevalence of CI which is consistent with the 25–40% reported elsewhere [28–30]. The pooled HAC positive rate was three times higher for those who screened positive for CI. This HAC rate is similar to a previous report for patients with dementia [24] recorded without screening. In this study screening for CI has identified more patients at risk so may be an important HAC reduction strategy.

The DCHP intervention required CI screening for all older patients on admission, an education program for clinical/non-clinical staff in the selected wards, the appropriate use of the CII and carer involvement with the aim of reducing the rate of HACs. We demonstrated that cognitive screening can be increased and maintained over a 40-week period and that the use of the CII, an important aid to facilitate clinical/non-clinical staff involvement, was found to be acceptable to patients and families. There was not direct measure of carer involvement. An improvement in staff satisfaction has been reported in separate papers [25, 32].

In patients who screened positive for CI, there was no significant change in the HAC rate as a result of the DHCP intervention (41.5% vs 43.2%). A pre-post study reported in 2023 using an adaptation of the DCHP and the same configuration of HACs was also negative adding weight to the possibility that this intervention does not have the intended effect on HACs [33]. Similar studies testing in-hospital multimodal interventions to reduce risk and subsequent complications, such as falls, have also failed to produce positive results [34].

It remains difficult to determine if the lack of effectiveness is a failure of the intervention, a failure of implementation or a failure of the measures of outcome. The DCHP has a number of characteristics that were consistent with a readiness for implementation [22]. The DCHP intervention had been used in varying hospital contexts—public, private city and regional. The involvement of people with dementia and their families in its development [9], recognition from the national dementia consumer group [35] and national government funding demonstrated stakeholder engagement and a system need. The use of validated screening tools, the programs perceived benefit by clinicians, and reports of improved staff satisfaction were also expected to support successful implementation. It was also expected that, a single national project officer, local fulltime project leads at each site, and the development of local clinical champions supported by a national education program delivered by the same investigators would support successful implementation. Despite the above, the degree to which the intervention was implemented as prescribed in the protocol and the number of patient 65 and over in participating wards as a percent of the total hospital population 65 and over varied significantly across sites (Supplementary Table 9).

The outcome measure of HACs is reliant on coding for CI which is based on information identifying CI in medical records. Across the intervention period, the number of patients who were screen negative for CI but coded for CI based on the medical record reduced suggesting that as the study progressed the accuracy of screening and its documentation improved.

The differences in outcomes likely reflect additional factors beyond patient demographics that could influence the effect of the intervention. For example, there may have been a general increase in the awareness of the needs of older people as a result of the DCHP resulting in better recognition of the potential risks of in hospital care for this large population [36].

None of the hospitals had retrospective data on rates of CI so it was necessary to collect this data during the training period which then became the control. Prior to the control period nursing staff were likely to be aware of some strategies to assist those with obvious dementia but it is known that they are often not aware of this deficit by observation alone. It is conceivable that screening in the control period would identify more patients with CI for whom they could apply the strategies they knew possibly reducing HAC rates prior to the introduction of the DCHP. This was a necessary compromise because there was no accurate retrospective hospital record data for CI and associated HAC rates because two thirds of patient with CI would have been missed [37]. In addition, it is difficult to engage staff in screening if it is not linked to any outcome and the timelines set by the funding body were constrained. Limiting training to only 40% of target staff before intervention data collection and completing training in the intervention period would have mitigated the effect of the contamination. The potential impact of the training contamination would be to reduce the likelihood of seeing a positive change in the intervention period. This potential type 2 error is noted but supports the conclusion that the significant difference in HAC rates reported is real.

A further limitation relates to CI capture: as cognitive impairment includes delirium, which is also an outcome when not present at admission. Delirium, as an outcome measure in this study, would require staff to have not recorded delirium on admission or when screening. Delirium recorded at a later stage would suggest an escalation in the symptomology so it was decided that the retention of delirium as an outcome measure was appropriate. It is expected that the HAC rates will vary by each complication, and future research with a breakdown by individual HAC, in addition to composite measures, is warranted.

There were 11,000 patients but only four hospitals which could lead to confounding through unbalanced site characteristics. One site had fewer males and another the population was slightly older, but the sites were relatively balanced. (Supplementary Table 6). As the site were not randomised there is increased risk of bias.

Common barriers to implementation reported by the project teams [26] included competing priorities for staff time for both education and screening, difficulty introducing the Clock Drawing Test in addition to usual screening, ward closures and ward leadership staff change and organization change fatigue. The timelines set by the federal government funding body and the reality that the systems to be changed were state government funded added to the complexity. This was reflected in the significant implementation variability across sites. Despite these complexities, while not achieving our pre-specified targets for our implementation strategies, we achieved moderate pooled rates for screening (70%), education for nurses (70%) and the CII usage(70%) [38]. The site that achieved the highest implementation strategy rates demonstrated a 13.4% reduction in HACs in patient with CI while the site with the least successful implementation strategy demonstrated a 12.4% increase (Supplementary Table S9).

The Australian health care system is funded by the National and State and Territory governments. Hospital services are State and Territory responsibilities with each jurisdiction having independent responsibility for hospital process and governance. The DCHP rollout was an Australian National Government project to implement change in the care of patients with CI across multiple independent jurisdictions. This engagement across diverse health care systems required flexibility within the protocol that is not desirable in research.

This study has demonstrated that the DCHP intervention can be implemented in four different hospitals nationally. If hospitals are to reduce HACs they need to identify at risk groups such as those with CI. Screening of CI is currently applied inconsistently. Once CI is identified all those who interact with the patient need to be alerted, including nurses, porters, catering staff and carers. It is for these reasons the DCHP intervention including the CII were developed.

After the completion of this study, Australia changed its National Safety and Quality Health Services Standards [39] to require all patients aged 65 years or over, or patients of any age at risk of delirium, to be screened for CI on admission. There is now an opportunity to have accurate baseline data for HACs secondary to CI in a hospital population. This will enhance future evaluations of programs like the DCHP. Further study is needed to understand reasons for variation in implementation and the impact of this variation on patient outcome measures.

## Conclusions

The DCHP can be feasibly implemented in a range of acute hospital settings. A small but significant reduction in HAC rates was seen in all participating older patients irrespective of cognitive status. The pooled results did not show a HAC reduction in patients with CI possibly because of site implementation variability. Hospital interventions to reduce HACs in older patients require rigorous implementation and evaluation.

## Supplementary Information

Supplementary Material 1.

## Data Availability

The datasets used and/or analysed for this study are available from the corresponding author on reasonable request.

## Abbreviations

CI: Cognitive Impairment
CII: Cognitive Impairment Identifier
DCHP: Dementia Care in Hospitals Program
DRGs: Diagnostic Related Groups
HAC: Hospital Acquired Complication
RR: Risk Ratio
UTI: Urinary Tract Infection
