# Supplementary material for: The impact of the Dementia Care in Hospitals Program on hospital acquired complications – a non-randomised stepped wedge hybrid effectiveness-implementation study
Source: BMC Geriatr. 2024 Dec 2;24:986. doi: 10.1186/s12877-024-05548-3 (PMC11610119; doi:10.1186/s12877-024-05548-3)
Supplement: Supplementary file 1 — Supplementary Material 1. [file 12877_2024_5548_MOESM1_ESM.docx]

**Title:** The impact of the Dementia Care in Hospitals Program on Hospital Acquired Complications – a non-randomised Stepped Wedge Hybrid Effectiveness-Implementation Study

**Authors:** Mark W. Yates MB BS^1^, Kasia Bail PhD^2,^ Sean MacDermott PhD^3^, David Skvarc PhD^4^, Meredith Theobald BN^5^, Michelle Morvell BN^6^, Jessica C Jebramek BPsychSc Hons^7^, Ian Tebbut BSc Hons^8^, Brian Draper MD^9^, Henry Brodaty DSc^10^

**Supplementary Table 1** Cognitive screening tests used across the four hospitals

| **Tool** | **Abbreviations** | **Criteria for positive CI screen** | **Used by Site** | **Reference** |
| --- | --- | --- | --- | --- |
| **Abbreviated Mental Test** | AMT | Score ≤ 7 | A | Hodkinson (1) |
| **Mini-Cog** |  | Recall 1 or 2 of 3 items and abnormal Clock Drawing; or recall of 0 of 3 words. | D | Borson (2) |
| **Abbreviated Mental Test Score 4*** | AMT4 | Score of 3 or less | Band C | Swain (3) |
| **Clock Drawing Test^** | CDT | Not all clock numbers present, spaced unevenly, or hands pointing to incorrect time. | B, C and D | Scanlan (4) |
| *Only used in conjunction with CDT  ^Used in conjunction with either AMT4 or MiniCog | | | | |

1. Hodkinson HM. Evaluation of a mental test score for assessment of mental impairment in the elderly. Age and Ageing. 2012;41(suppl_3):iii35-iii40.

2. Borson S, Scanlan JM, Watanabe J, Tu S-P, Lessig M. Improving identification of cognitive impairment in primary care. International Journal of Geriatric Psychiatry. 2006;21(4):349-55.

3. Swain DG, Nightingale PG. Evaluation of a shortened version of the Abbreviated Mental Test in a series of elderly patients. Clinical Rehabilitation. 1997;11(3):243-8.

4. Scanlan JM, Brush M, Quijano C, Borson S. Comparing clock tests for dementia screening: naïve judgments vs formal systems—what is optimal? International Journal of Geriatric Psychiatry. 2002;17(1):14-21.

**Supplementary Table 2.** Study Participant characteristics.

| **Hospital** | **Possible Population**  **65+** | **Eligible Study Population (% of pooled study population)** | **Study Population mean age (range and SD)** | **Study Population Sex Male %** | **Study Population % Surgical**  **Ward** |
| --- | --- | --- | --- | --- | --- |
| **A** | 9048 | 5079 (30.2) | 78.9 (50-104, 8.25) | 46.2 | 27.4 |
| **B** | 8204 | 4130 (24.5) | 76.4 (50-102, 8.02) | 47.9 | 22.5 |
| **C** | 17162 | 3554 (21.1) | 77.8 (50-103, 8.88) | 42.8 | 28.9 |
| **D** | 13402 | 4026 (24.2) | 77.2 (50-106, 8.27) | 49.9 | 31.5 |
| **Pooled** | 47816 | 16789 (100) | 79.2 (50-106, 8.7) | 46.8 | 27.5 |

**Supplementary Table 3.** Staff Training by craft group

| **Work Category** | **Medical** | **Nursing** | **Allied Health** | **Non- Clinical^** | **Other** | **Total** |
| --- | --- | --- | --- | --- | --- | --- |
| Number of staff | 411 | 1261 | 302 | 555 | 58 | 2587 |
| Number of staff trained | 247 | 916 | 211 | 323 | 51 | 1748 |
| Average staff trained | 60.1% | 72.6% | 69.9% | 58.2% | 87.9% | 67.6% |
| Range of staff trained in the target wards | 50.4% -  100.0% | 57.5% -  83.0% | 19.4% -  100.0% | 40.4% -  98.4% | 82.9%-  100.0% | 61.3% -  74.2% |

^ Non-clinical staff included ward clerks, food and domestic staff, porters and cleaners)

**Supplementary Table 4.** Screening rate as a proportion of the target population by period and proportion of those with CI

| **Period** | **Eligible Study Population**  **(from participating wards)** | **Study Sample**  **(% of the eligible study population screened)** | **Population with CI**  **(% of study sample)** |
| --- | --- | --- | --- |
| **Control** | **3324** | **1970 (59%)** | **905 (45.9%)** |
| Intervention Period (T1) | 4172 | 2914 (69.8%) | 1,146 (39.3%) |
| Intervention Period (T2) | 3138 | 2144 (63.3%) | 795 (37.1%) |
| Intervention Period (T3) | 3017 | 2199 (72.9%) | 720 (32.7%) |
| Intervention Period (T4) | 3138 | 2082 (66.3%) | 712 (34.2%) |
| **Total Intervention Period** | **13465** | **9339 (71%)** | **3373 (36.1%)** |
| Total study sample | 16789 | 11309(67.2%) | 4278 (37.8%) |

Note –

Eligible Study Population = population over the age of 65 in the 20 target wards during the period of the study.

Study Sample = population in the 20 target wards who were screened.

CI population = the population who screened positive for cognitive impairment.

**Supplementary Table 5.** The CI screen positive offered the CII by time period.

|  | **Intervention Period (T1)** | **Intervention Period (T2)** | **Intervention Period (T3)** | **Intervention Period (T4)** | **Total** |
| --- | --- | --- | --- | --- | --- |
| Offered CII (% if positive) | 601(70%) | 506 (80.1%) | 388 (63.9%) | 412 (64.5%) | 1907 (69.8%) |
| Total Screen Positive | 858 | 628 | 607 | 639 | 2732* |

Note - * ^The CI rates for CII display had missing data in each period, as it was often hard to ascertain CII use due to short stays, weekend admissions, and overnight when project officers weren’t able to visually sight the bedside CII

**Supplementary Table 6.** Study population by hospital site.

| **Site** | **Possible patient pool - all “older patients” in the participating hospitals** | | | **Eligible population - all of the possible patients in the participating wards (% of the possible patients)** | | | **Study sample population - all those screened (%)** | | | **DCHP sample population who Screened +ve for CI (%)** | | |
| --- | --- | --- | --- | --- | --- | --- | --- | --- | --- | --- | --- | --- |
|  | Control | Intervention | Total | Control | Intervention | Total | Control | Intervention | Total | Control | Intervention | Total |
| A | N/A | N/A | 9048 | N/A | N/A | 5079 (56.1%) | 275 | 2621 | 2896 (57%) | 125 (45%) | 711 (27%) | 836 (29%) |
| B | N/A | N/A | 8204 | N/A | N/A | 4130 (50.3%) | 860 | 2321 | 3181 (77%) | 474 (55%) | 1012(44%) | 1486 (47%) |
| C | N/A | N/A | 17162 | N/A | N/A | 3554 (20.7%) | 528 | 1405 | 1933 (54%) | 136 (25%) | 602 (43%) | 738 (38%) |
| D | N/A | N/A | 13402 | N/A | N/A | 4026 (30.1%) | 307 | 2992 | 3299 (82%) | 170 (55%) | 1048 (35%) | 1218 (37%) |
| Pooled | N/A | N/A | 47816 | 3324 | 13465 | 16789 (35%) | 1970 (59%) | 9339 (69%) | 11309 (67%) | 905 (46%) | 3373 (36%) | 4278 (38%) |

**Notes** - Site C lowest eligible population as a proportion of the possible patient pool - 21% compared to the highest proportion at Site A 56%. This may have had impact on the implementation of the DCHP which leverages off an “all of hospital” engagement because many clinical and non-clinical staff working across multiple wards.

**Supplementary Table 7.** Percentage HAC positive by screening status and hospital site

|  |  | **Control Period** | | **Intervention Period** | |  |  |
| --- | --- | --- | --- | --- | --- | --- | --- |
| **Hospital** | **Cohort** | **HAC** | **%** | **HAC** | **%** | **Change in HAC rate** | **P value/significance** |
| **Site A** | CI | 60 | 48.0% | 246 | 34.6% | -13.4% | P = .004** |
|  | No CI | 31 | 20.7% | 298 | 15.6% | -5.1% | P = .10 |
|  | Combined | 91 | 33.1% | 544 | 20.8% | -12.3 % | P <.001*** |
| **Site B** | CI | 172 | 36.3% | 437 | 43.2% | + 6.9% | P = .011* |
|  | No CI | 62 | 16.1% | 222 | 17.0% | +0.9% | P = .67 |
|  | Combined | 234 | 27.2% | 659 | 28.4% | +1.2% | P = .50 |
| **Site C** | CI | 59 | 43.4% | 336 | 55.8% | +12.4% | p = .008** |
|  | No CI | 86 | 21.9% | 157 | 19.6% | - 2.3% | P = .35 |
|  | Combined | 145 | 27.5% | 523 | 37.2% | +9.7% | P = .001** |
|  | CI | 85 | 50.0% | 451 | 43.0% | -7% | P = .08 |
| **Site D** | No CI | 29 | 21.2% | 342 | 17.6% | -3.6% | P = .28 |
|  | Combined | 114 | 37.1% | 793 | 26.5% | -10.6% | P = .001** |
| **Pooled** | CI | 376 | 41.2% | 1470 | 43.2% | +2% | P=.28 |
|  | No CI | 208 | 19.6% | 1049 | 17.6% | -2% | P = .11 |
|  | Combined | 584 | 29.6% | 2519 | 27.0% | -2.6% | P = .01* |
| HAC = Hospital acquired complications  CI = Cognitive Impairment  Change in HAC is Relative Risk  P values are unadjusted | | | | | |  |  |

**Supplementary Table 8** Secondary Outcomes Measures

| **Patient Quality of Life** - MacDermott S, Yates M, Theobald M, Morvell M, Mohebbi M, West E, Jebramek J, Watts JJ (2017). National Rollout and Evaluation of the Dementia Care in Hospitals Program (DCHP), Ballarat: Victoria (Prepared for the Department of Health). www.bhs.org.au/services-and-clinics/dementia-care-in-hospitals-program/ |
| --- |
| **Intervention Cost** – MacDermott S, Yates M, Theobald M, Morvell M, Mohebbi M, West E, Jebramek J, Watts JJ (2017). National Rollout and Evaluation of the Dementia Care in Hospitals Program (DCHP), Ballarat: Victoria (Prepared for the Department of Health). www.bhs.org.au/services-and-clinics/dementia-care-in-hospitals-program/ |
| **Staff Satisfaction and Knowledge**- Murray ME, Wong Shee A, West E, Morvell M, Theobald M, Versace V, et al. Impact of the Dementia Care in Hospitals Program on acute hospital staff satisfaction. BMC Health Services Research. 2019;19(1):680. |
| **Total antipsychotic usage per admission; Anticholinergic load per admission -** not reported |
| **In-hospital falls and mortality –** not reported |

**Supplementary Table 9** Measures of Implementation by site.

| **Intervention strategy** | **Site A** | **Site B** | **Site C** | **Site D** | **Source** |
| --- | --- | --- | --- | --- | --- |
| **Screening for CI by site** | 57% | 77% | 54% | 82% | Table S6 |
| **Staff Education** | 69% | 62% | 59% | 43% | National Report |
| **CII usage as a % of CI positive** | 48.3% | Incomplete* | 35% | 47.5% | National report |
| **Service Engagement** | 56.3% | 50.5% | 20.7% | 30.1% | Table S6 |
|  |  |  |  |  |  |
| **Change in HAC rate in those with CI** | **-13.4%** | **+6.9%** | **+12.4** | **-7%** |  |

- *The CII use as verbally reported by the project officer was low
- ** The four intervention outcomes were ranked from 4 for best to 1 for worst for a total maximum score of 16
- Service Engagement – The proportion of all older people in the hospital who were included in the intervention. This is reported as the eligible population (the number of older patients in participating wards) as a % of all older patients in the hospital
